# Fabrication of porosity-controlled polyethylene terephthalate porous materials using a CO_2_-assisted polymer compression method

**DOI:** 10.1039/c7ra12184a

**Published:** 2018-01-15

**Authors:** T. Aizawa

**Affiliations:** National Institute of Advanced Industrial Science and Technology, Research Institute for Chemical Process Technology 4-2-1 Nigatake, Miyagino-ku Sendai 983-8551 Japan t.aizawa@aist.go.jp

## Abstract

The objective of this study is to fabricate porosity-controlled polyethylene terephthalate porous materials using a CO_2_-assisted polymer compression (CAPC) method. In a previous study, the CAPC method was used to fabricate porous polymer materials by compressing fabric sheets in the presence of CO_2_. However, the controllability of the porosity was not clear in the previous study. In this study, it is shown that the porosity of porous polymer materials could be easily controlled by adjusting the operating conditions of the CAPC method, using polyethylene terephthalate (PET) nonwoven fabric sheets. Using mercury porosimetry, a decrease in the porosity induced by compression accompanied by a decrease in the pore size is demonstrated. Scanning electron micrographs strongly indicate the plasticization of PET fibers by CO_2_.

## Abstract 2

Porosity-controlled polyethylene terephthalate porous materials can be easily fabricated by using a CO_2_-assisted polymer compression (CAPC) method.

## Graphical abstract

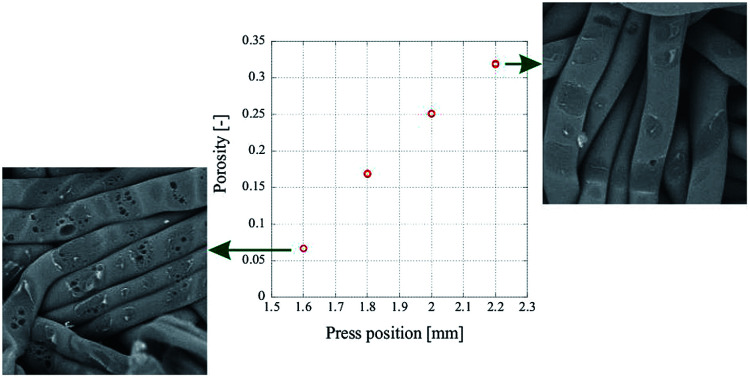

## Introduction

Since CO_2_ is non-toxic, it is attractive as an alternative solvent for green chemistry.^1^ In terms of substituting organic solvents, it can be applied in processes such as chemical reactions and extraction.^2–5^ Particularly, when used in extraction, CO_2_ has high penetrability and solubility that can be controlled by changing the density, and the concentration process is simultaneously achieved while performing the separation. Therefore, CO_2_ is expected to be applied in various extraction processes.^6,7^ The sustainability of CO_2_ is also suitable for its use in the field of pharmaceuticals.^8^ Processes using CO_2_ for material synthesis have also been proposed.^9,10^ Studies related to the utilization of CO_2_ for polymer reaction engineering have also been reported.^11,12^ For example, a polymer synthesis process using CO_2_ as a chemical reaction medium has been proposed.^13,14^ Exploiting the decrease in the solubility of the polymer with increasing degree of polymerization, synthesis of polymers with uniform particle sizes is possible. Using CO_2_, development of a fine particle manufacturing process using a synthesized polymer has also been proposed.^15,16^ As methods for producing fine particles, spraying CO_2_-dissolved polymer, spraying CO_2_-dissolved molten polymer, and spraying polymer-dissolved organic solvent to CO_2_ are proposed. The role of CO_2_ varies depending on its use as a solvent, plasticizer, or anti-solvent. A process for fabricating a foamed polymer using CO_2_ in the gaseous state under normal conditions has also been proposed.^17–19^ Molding a polymer using only CO_2_ prevents contamination of the polymer and is particularly effective for their application in pharmaceutical fields where safety is of utmost importance. Moreover, as impurity-free polymers are excellent for recycling, the process also reduces the environmental impact.

CO_2_-assisted polymer compression (CAPC) is a technique developed for the facile fabrication of porous polymer materials by compressing fibrous sheets in CO_2_.^20^ CAPC is a suitable process for plasticizing the surface of a polymer using liquid CO_2_ at room temperature and promoting the adherence of the resin fibers with each other by compression, to produce porous polymer materials. The method could be adapted to various polymers that can be plasticized by liquid CO_2_. Pores are generated by an increase in the volume of the void between the fibers. In addition, by introducing CO_2_ as a vapor pressure gas and using the piston not only for compressing the polymer but also to liquefy CO_2_, the requirement of a high-pressure pump was eliminated and a very simple apparatus configuration was realized. The development of a new process was reported in a previous article; however, the relationship between the operating conditions of the process and the physical properties of the formed porous polymer materials was not established.

For quantitative analysis, it is necessary to procure uniform nonwoven fabrics using polymers with known physical properties. Therefore, polyethylene terephthalate (PET) nonwoven fabric with a uniform fiber diameter custom-produced using a specific pellet was used in this study. Using this sheet as a prototype, the operating conditions of the process were varied and the possibility to control the porosity of the porous polymer materials was assessed. This is the first time that the process control of the CAPC method is demonstrated.

## Experimental

**Fig. 1:**
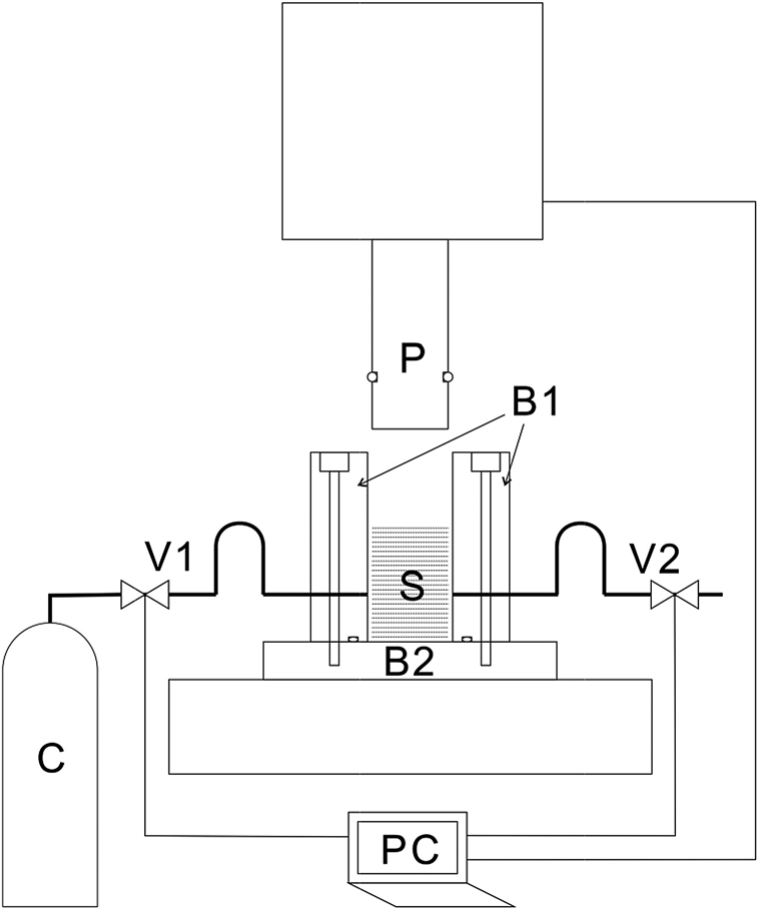
Schematic illustration of the cross-section of the high-pressure vessel. B1: body of the high-pressure vessel, B2: base of the high-pressure vessel, C: CO_2_ cylinder, P: piston, PC: laptop PC, S: sample, V1: intake valve, and V2: exhaust valve.

Details of the process are described in a previous publication^20^ and only a brief outline of the process is provided here. A cross-sectional view of the piston and the press unit is shown schematically in Fig. 1. The press machine is JP-1504 manufactured by Janome Sewing Machine Co., Ltd. The piston (outer diameter, 19.5 mm) and the pressure vessel (inner diameter, 20.0 mm) were custom-designed and manufactured. Stainless-steel tubes were connected to the pressure vessel for the introduction and exhaustion of CO_2_. Valves were connected to both the tubes. The piston and the valves were controlled by a laptop personal computer (PC), and after setting the sample, the piston and the valves were operated at specified timings, and the processing was fully automated until completion.

A PET nonwoven fabric with an average fiber diameter of 8 μm was custom-made by Nippon Nozzle Co., Ltd. using a melt-blown method with TK3 pellets (Bell Polyester Products, Inc.). The basis weight of this nonwoven fabric is about 30 g m^−2^, and it was cut using a punch of 18.0 mm diameter. Regarding punching accuracy, the entire surface of the printer paper was punched out; the area per sheet was calculated from the weight of the original paper and the weight per sheet punched out, and the 18.0 mm diameter of the punched-out sheet was verified.

First, 32 pieces (0.245 to 0.248 g), 64 pieces (0.492 g), or 96 pieces (0.738 g) of circularly-cut nonwoven fabric sheets were dropped from the top of the pressure vessel. After setting the samples, the piston was lowered to a predetermined position (introduction position), and the air in the container was replaced with CO_2_ through valve operations. Subsequently, CO_2_ was introduced by vapor pressure. After that, the piston was lowered to a predetermined pressing position (press position) and held for 10 s. Then, CO_2_ was exhausted by opening the exhaust valve. The sample was retrieved after raising the piston. Since the introduction position of CO_2_ and the press position differed for each experiment, the distance from the bottom of the pressure vessel each time is clearly indicated. The press time is 10 s unless otherwise stated. Experiments with 4 s pressing time were also attempted to verify the dependence of the porosity on the press time, which is clearly stated during related discussions.

The difference between introduction position and press position influences the amount of liquefied CO_2_. When the vessel is filled with gaseous CO_2_ at its vapor pressure at the introduction position and then partially liquefied by lowering the piston to the press position, the following relationship is established:

1 *d*_g_*V*_i_ = *d*_l_*V*_l_ + *d*_g_(*V*_p_ − *V*_l_),

where *V*_i_ and *V*_p_ represent the volume of the vessel at the introduction and press position, respectively; *d*_g_ is the density of the gas at its vapor pressure, *d*_l_ is the density of the liquid, and *V*_l_ is the volume of the liquid at the press position. The volume *V*_l_ of the liquid is derived as follows:2
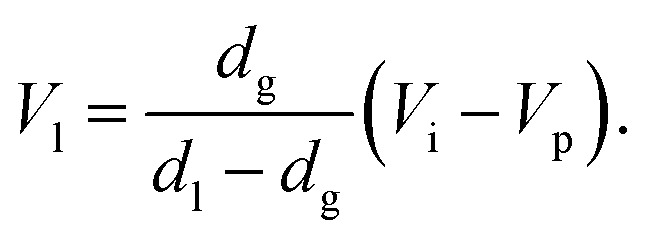


Since (*V*_i_ − *V*_p_) is decreased by lowering the piston, the amount of liquefied CO_2_ after the movement of the piston by 0.1 mm is evaluated to be 0.016 mL at 25 °C, using *d*_g_ = 0.243 g mL^−1^, *d*_l_ = 0.711 g mL^−1^ and the vessel inner diameter of 20.0 mm.

In this study, the method used for controlling the piston was changed from that of the previous study.^20^ In the previous study, the piston was slowly lowered by controlling the load. Since it takes time to process by this method, the piston was moved by position control that is faster and practical in this study. The set value of the moving speed of the piston was 20 mm s^−1^; however, the actual speed is speculated to be slower than this, owing to deceleration near the end and beginning of the movement of the piston.

As for the evaluation of the experimental results, since the density of this polymer is disclosed as 1.34 g mL^−1^ by the datasheet of PET pellet (Bell Polyester Products, Inc.), the thickness in the case of a solid without a void can be easily calculated by its weight and area. Therefore, the thickness at the center of the porous polymer material after the treatment was measured using a micrometer screw gauge, and the difference between the theoretical thickness and the actual thickness was considered as the total area of the pore. Using this, the porosity was calculated. The reason for measuring the thickness at the center part is that the inner diameter of the pressure vessel was 20.0 mm and the diameter of the set nonwoven fabric was 18.0 mm, so that the part where not all the nonwoven fabrics overlap is at the periphery. In theory, all the nonwoven fabrics are overlapped within the radius of 8.0 mm from the center (central 16.0 mm diameter); therefore, the thickness of the center part was measured. When the material is cylindrical with 18.0 mm diameter and no void, the heights of the PET cylinders are calculated as 0.722 mm at 0.246 g, 1.44 mm at 0.492 g, and 2.16 mm at 0.738 g. The difference between the measured values and above calculated values is the area of the pore.

Pore size distribution was evaluated by mercury porosimetry (Micromeritics AutoPore IV 9500). The produced porous polymer materials were incompletely overlapped in the peripheral portion, and the peripheral portion was cut off using a cutter and only the central portion was loaded in the cell for performing the measurement. As the adsorption of water by the test piece influences the time of initial vacuuming of the mercury porosimetry cell, the sample was dried overnight in an oven at 80 °C. Porosity could be measured by mercury porosimetry; however, as specified in the user manual, the accuracy of the volume is 1% of the full volume of the mercury porosimetry cell. The fluctuation in the blank calibration data indicates this accuracy, and this fluctuation is larger than the fluctuation in the porosity of the produced material. For this reason, height measurements were carried out to determine the porosity, and mercury porosimetry was used only to determine the pore size distribution.

The surfaces of the central layers of the porous polymer materials were observed using a scanning electron microscope (Hitachi High-Technologies TM-1000). To observe the state of the fiber at the joint, a notch was made at the end of the central layer of the porous polymer material with a cutter and it was manually pealed into two units before observing its surface with a scanning electron microscope (SEM). For comparison, native samples before treatment and samples only exposed to liquid CO_2_ were also observed. The exposure to liquid CO_2_ was carried out by loading one sheet of the nonwoven fabric into the pressure vessel. Since the movement of the piston was same, it facilitated both the exposure of the sample to liquid CO_2_ (10 s) and rapid escape of CO_2_. As only one nonwoven fabric was loaded, there was sufficient space between the bottom of the pressure vessel and the piston, and the nonwoven fabric was not compressed even when the piston was lowered to the fullest extent.

## Results and discussion

**Fig. 2:**
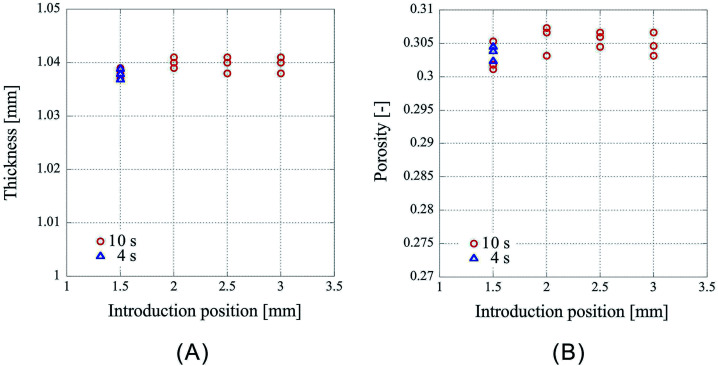
Characteristics of the porous polymer material fabricated from 32 PET nonwoven fabric sheets using different CO_2_ introduction positions. (A) Central thickness of the sample, (B) porosity. Circles: pressing time is 10 s, triangles: pressing time is 4 s.

First, the influence of the introduction position of CO_2_ on the porosity was examined. The results obtained for 32 nonwoven fabrics placed in the high-pressure vessel, with the press position fixed at 1.10 mm, and the introduction position of CO_2_ set to 1.50 mm, 2.00 mm, 2.50 mm, 3.00 mm, respectively, are represented as circles in Fig. 2. The experiments were conducted thrice under each condition. Fig. 2A shows the thickness of the center portion of the porous polymer materials, and Fig. 2B shows the porosity calculated from the thickness. CO_2_ partially liquefies owing to compression due to the movement of the piston from the introduction position to the press position, so that the change in the introduction position would affect the amount of CO_2_ that is liquefied; however, for the press time used, there was no influence on the porosity of the material within the range of positions studied. Interestingly, the processed porous polymer materials were found to be slightly thinner than the pressed position, which implies that they shrunk owing to desorption of CO_2_.

The experiments were also conducted with another press time. The circles in Fig. 2 represent the results for the pressing time of 10 s. The experiments with the pressing time of 4 s were carried out in the case of the CO_2_ introduction position of 1.50 mm, and the results are represented by triangles in Fig. 2. The weight of the sample in Fig. 2 is 0.246 g or 0.247 g. Fig. 2A shows that the thicknesses of the samples (indicated by circles and triangles) overlap, however, because the weights of the overlapped samples are different, they do not overlap in the porosity graph (Fig. 2B). From these results, it is clear that there is no difference in the sample characteristics between the press times of 4 and 10 s. In this study, all the subsequent processes described henceforth were performed at the press time of 10 s.

**Fig. 3:**
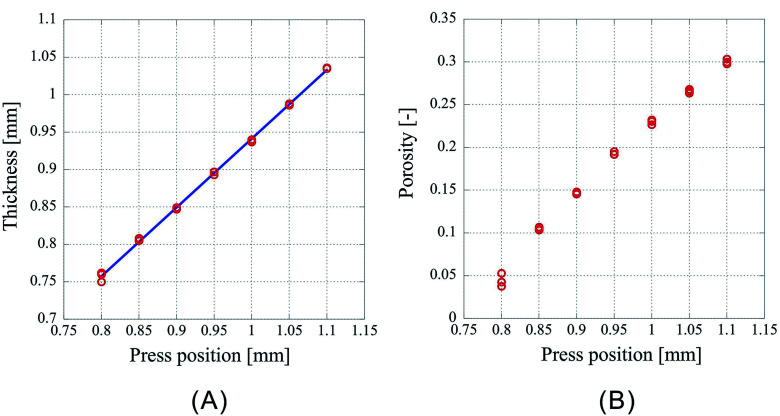
Characteristics of the porous polymer material fabricated from 32 PET nonwoven fabric sheets. (A) Central thickness of the sample, (B) porosity.

Next, the results of fixing the CO_2_ introduction position to 1.50 mm and lowering the press position such that the press positions are 1.10 mm, 1.05 mm, 1.00 mm, 0.95 mm, 0.90 mm, 0.85 mm, 0.80 mm are shown in Fig. 3. Fig. 3A shows the thickness of the center of the porous polymer materials, and Fig. 3B shows the porosity. The experiments were conducted thrice under each condition. As the pressing position is lowered, the amount of liquefied CO_2_ should increase. However, this effect may be neglected considering the results from the previous experiment in which the introduction position was changed. As the exposure time of the polymer to liquid CO_2_ is same, the porosity change is solely due to the change in the pressing position. From the plots of the press position and press thickness (Fig. 3A), it is clear that the samples after pressing are shrunk irrespective of the press position. The press position and the thickness of the processed samples appear to be in a linear relationship. This is very well supported by the correlation coefficient of 0.99948 obtained by the linear fitting of the data using the least squares method. This indicates that the thickness achieved after pressing can be predicted from the pressing position and that the porosity of the porous polymer materials can be controlled by adjusting the operating condition of the process. Indeed, the ability to arbitrarily control the porosity by an external factor such as the press position is a remarkable merit of the CAPC method.

**Fig. 4:**
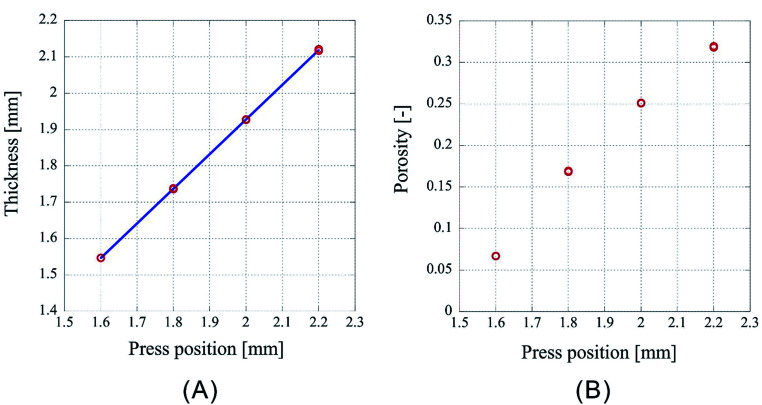
Characteristics of the porous polymer material fabricated from 64 PET nonwoven fabric sheets. (A) Central thickness of the sample, (B) porosity.

**Fig. 5:**
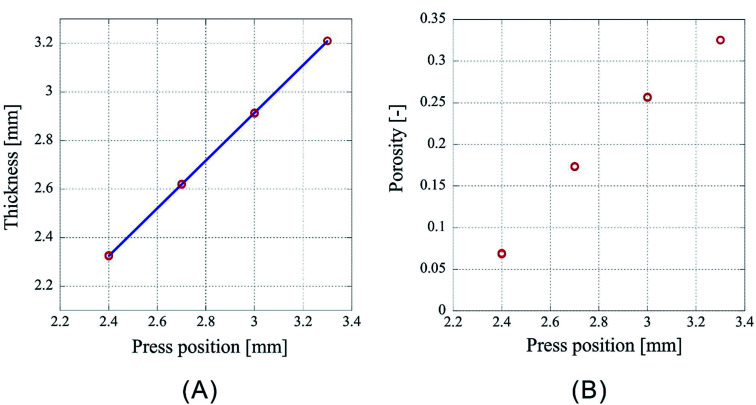
Characteristics of porous polymer material fabricated from 96 PET nonwoven fabric sheets. (A) Central thickness of the sample, (B) porosity.

However, as Fig. 3 is only the result obtained when the amount of sample is limited, the experiment was carried out by setting the number of sheets to 64 and 96, which is an increase by a factor of two and three, respectively, than the previous experiment. As the experimental conditions for 64 sheets, the introduction position of CO_2_ was fixed at 3.00 mm and the pressing positions were set to 2.20 mm, 2.00 mm, 1.80 mm, and 1.60 mm. In case of 96 sheets, the introduction position of CO_2_ was fixed at 4.50 mm and the pressing positions were set to 3.30 mm, 3.00 mm, 2.70 mm, and 2.40 mm. Experiments were conducted twice under each condition for both cases and the results are shown in Fig. 4 and 5. In both cases the finished samples were slightly thinner than the press position, which suggests their shrinkage. The linear relationship between the thickness of the processed sample and the press position observed in case of 32 sheets was also observed for these two cases. The correlation coefficients are 0.99998 for 64 sheets and 0.99999 for 96 sheets, implying that the linear fittings are extremely satisfactory. These results suggest that when a certain amount of sample is loaded, it is possible to control the porosity of the porous polymer materials by the relationship between press position and the thickness achieved after pressing.

However, according to the results, the slopes of the 32, 64, and 96 fittings are different. The slope corresponding to 32 sheets is 0.919, and those for 64 and 96 sheets are 0.953 and 0.982, respectively. It is not clear whether this is experimental error or happens due to specific reasons. The following reasons are conceivable: there may be a difference in the mechanism of compression of a nonwoven fabric close to a hard surface, such as a piston or pressure vessel, and that of a nonwoven fabric sandwiched between adjacent nonwoven fabrics on both sides. Because the ratio of either is different depending on the sample thickness, the amount of compression may depend on the sample thickness. This will be clarified in the future by detailed analysis of data of compression tests with different types of materials when they become available.

**Fig. 6:**
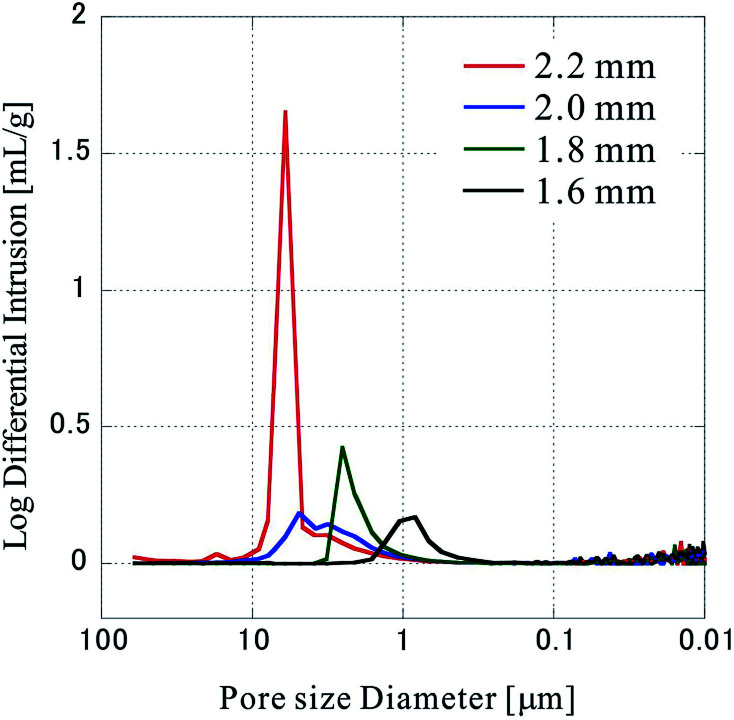
Pore size distribution in the sample. The pore size distribution was evaluated by mercury porosimetry.

Next, the pore size distribution of the porous polymer materials fabricated from 64 sheets was measured by mercury porosimetry, and the results are shown in Fig. 6. The measurements were performed thrice with the press positions of 2.20 mm, 1.80 mm, 1.60 mm, and four times with a press position of 2.00 mm, all of which show almost the same distribution at each position. It became clear that the pore size distribution shifts to a smaller size range as the press position is lowered (the compression ratio increased). This is reasonable considering that nonwoven fabric sheets originally have large pores (void area), and that the pores become small according to the extent of compression. The distribution of the pore size at 2.00 mm is rather wider compared to those of samples fabricated at other press positions. As mentioned earlier, four samples were measured at 2.00 mm. Since all of them show the same tendency, it seems that a specific phenomenon occurs at the 2.00 mm press position.

**Fig. 7:**
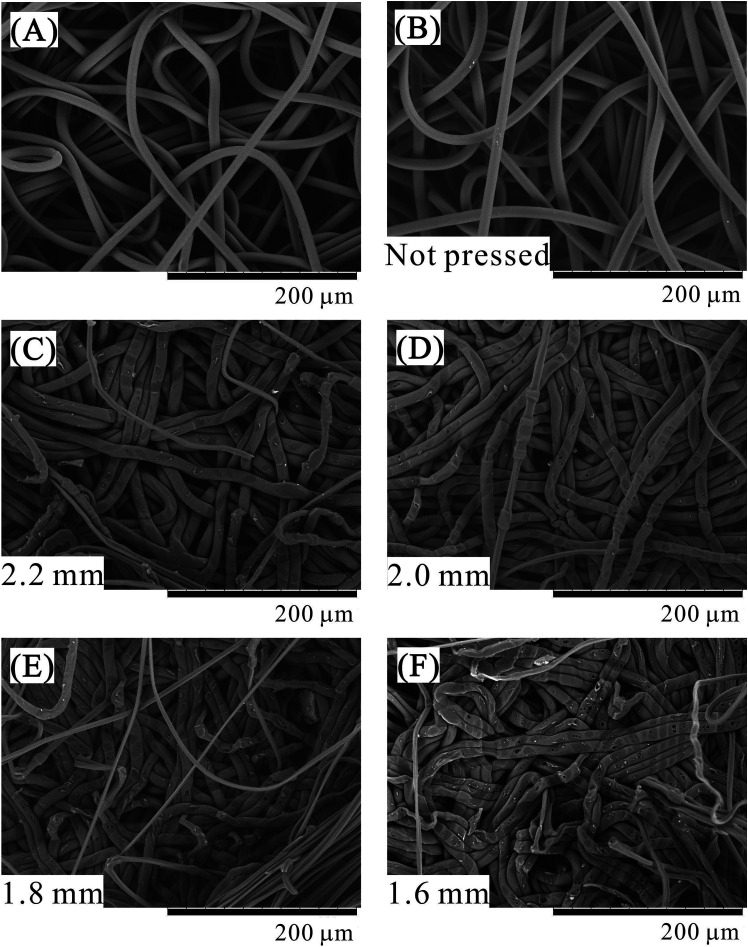
SEM images of the samples. (A) Sample before processing, (B) sample exposed to liquid CO_2_ without applying pressure. Porous polymer material fabricated from 64 PET nonwoven fabric sheets at different press positions, (C) 2.2 mm, (D) 2.0 mm, (E) 1.8 mm, (F) 1.6 mm.

The SEM images of the surface of the central nonwoven fabric sheet of porous polymer materials fabricated using 64 sheets are shown in Fig. 7. Fig. 7A shows the surface of the nonwoven fabric before treatment and Fig. 7B shows the surface of a single nonwoven fabric placed in a pressure vessel and exposed to liquid CO_2_ without pressing. In Fig. 7B, the CO_2_ introduction position of the piston is 3.00 mm and the press position is 1.60 mm, which is same as that used for 64 sheets at 1.60 mm pressing position. Fig. 7C–F show the SEM images of samples pressed in the presence of CO_2_, and pressed positions are clearly indicated in the figure. SEM is a surface observation method that is good for observing micropores with clear boundaries in a sliced sample. However, it is not suitable for observing pores generated by fiber voids because of their unclear boundaries. In these figures, a few extremely fine fibers that are not observed in the SEM of the native sample before treatment are observed. These fibers are stretched because of the pealing of the porous polymer material to observe the middle layer of the porous polymer material. The presence of the stretched fibers suggests that parts of the fibers are strongly adhered. As the press position is lowered, the nonwoven fabric sheets are compressed, and remarkable changes occur in their structure, as observed in the SEM images. First, there is no difference between the sample (A) before treatment and the sample (B) only exposed to CO_2_ without pressing. Next, in the sample pressed in the presence of CO_2_, as the press position is lowered, increased indentation due to pressing against the other fibers is observed. Further, in addition to the indentation, parts where the fibers spread sideways by being crushed are also conspicuous. Since the glass transition point of PET is 70 °C, it is unlikely that the glass transition point decreased to a value equivalent to room temperature in the presence of liquefied CO_2_. In this system, CO_2_ should dissolve in the amorphous regions of the polymer and cause its plasticization. As a further characteristic phenomenon, small bubbles become conspicuous on the fiber surface. The bubbles are frequently found in recessed portions where fibers overlap each other. It seems that CO_2_ dissolved in the polymer surface cannot be desorbed at a rapid speed due to the strong compression, and hence, CO_2_ formed bubbles on the fiber surface capped by other polymer fibers. Although this phenomenon was hardly observed for sample processed using the press position of 2.20 mm with a weak pressing force, it increased to 2.00 mm, 1.80 mm, 1.60 mm as the press position was lowered. As the exposure time of the polymer to liquid CO_2_ is the same, the contribution of the compression process to the generation of bubbles is significant. The presence of bubbles strongly suggests that CO_2_ is dissolved in the polymer and the polymer is plasticized.

A dent was also observed in the nonwoven fabric of the polyethylene (PE) and PET composite reported in the previous paper,^20^ but no spreading of fibers or bubbles was observed. It seems that the degree of plasticization is obviously larger in the PET nonwoven fabric used in this study than the nonwoven fabric used in the previous one (PE and PET composite). It is known that polymers are plasticized by the dissolution of CO_2_;^21–23^ therefore, the difference in the solubility^24–26^ of CO_2_ for each polymer is important. In this case, it seems that the solubility of CO_2_ in PET is larger than that in PE. According to Shieh *et al.*,^25^ the solubility of CO_2_ in PET and low-density polyethylene is 1.5 wt% and 0.4 wt%, respectively, at a pressure of 13.8 MPa and a temperature of 40 °C. Although there might be a difference between the supercritical and liquid states, it is suggested that PET is more likely to dissolve CO_2_ and undergo plasticization than PE.

## Conclusions

Production of porosity-controlled porous polymer material using a CO_2_-assisted polymer compression method is demonstrated for the first time using custom-made nonwoven fabric sheets with clear characteristic of PET pellets. The porosity formed by the void areas between the fibers could be controlled by controlling the press position. In the experiments using PET with an average fiber diameter of 8 μm, a linear relationship was established between the pressing position and the thickness of the porous polymer materials. Analysis by mercury porosimetry revealed that when the compression ratio was increased, the pore size distribution shifted to a smaller value, due to the compression of the void area between the fibers. In addition, SEM image analysis revealed that increasing the compression ratio increases the indentation of the fiber, and the formation of bubbles at the fiber surface owing to the increase in the confinement of CO_2_. This phenomenon strongly suggests plasticization of the PET fiber due to the dissolution of CO_2_ into the polymer.

## Conflicts of interest

There are no conflicts to declare.

## Supplementary Material
